# Ideotype breeding for crop adaptation to low phosphorus availability on extensive organic farms

**DOI:** 10.3389/fpls.2023.1225174

**Published:** 2023-07-18

**Authors:** Michelle Katherine Carkner, Xiaopeng Gao, Martin H. Entz

**Affiliations:** ^1^ Department of Plant Science, University of Manitoba, Winnipeg, MB, Canada; ^2^ Department of Soil Science, University of Manitoba, Winnipeg, MB, Canada

**Keywords:** ideotype breeding, nutrient cycling, ecological nutrient management, organic agriculture, phosphorus use efficiency, micronutrient bioavailability

## Abstract

Organic farming in extensive production regions, such as the Canadian prairies have a particularly difficult challenge of replenishing soil reserves of phosphorus (P). Organic grains are exported off the farm while resupply of lost P is difficult due to limited availability of animal manures and low solubility of rock organic fertilizers. As a result, many organic farms on the prairies are deficient in plant-available P, leading to productivity breakdown. A portion of the solution may involve crop genetic improvement. A hypothetical ‘catch and release’ wheat ideotype for organic production systems is proposed to (i) enhance P uptake and use efficiency but (ii) translocate less P from the vegetative biomass into the grain. Root traits that would improve P uptake efficiency from less-available P pools under organic production are explored. The need to understand and classify ‘phosphorus use efficiency’ using appropriate indices for organic production is considered, as well as the appropriate efficiency indices for use if genetically selecting for the proposed ideotype. The implications for low seed P and high vegetative P are considered from a crop physiology, environmental, and human nutrition standpoint; considerations that are imperative for future feasibility of the ideotype.

## 1 Introduction

### 1.1 Phosphorus challenge on organic farms

Phosphorus (P) management is a particular challenge for Canadian organic farms on the prairies. While most conventional farming systems heavily rely on inputs of synthetic nitrogen (N) fertilizers, organic farms often maintain N levels through growing legumes within the green manure and forage phase of a crop rotation. However, replenishing P is more difficult on organic farms as crop harvest removal of grain or biomass continues to shrink the soil nutrient reservoir (Morrison and Kraft, 1994). Several on-farm studies have reported low soil test phosphorus status on Canadian organic farms (Entz et al., 2001; Martin et al., 2007; Roberts et al., 2008; Knight et al., 2010) Entz et al. (2001) surveyed 14 organic farms in Manitoba, Saskatchewan, and North Dakota, USA, and reported an average soil test phosphorus of 15 kg P ha^-1^, which was substantially lower than the Manitoba average value for agricultural lands (> 20 kg P ha^-1^) (Entz et al., 2001). After 13 years of organic production at the Glenlea Long-term Rotation Study site in Manitoba, soil available P fractions rapidly declined in the organic forage rotation (Welsh et al., 2009; Carkner et al., 2020). Additionally, low soil test phosphorus has also been reported on organic farms which lack livestock in Saskatchewan (Knight et al., 2010), and organic dairy farms in Ontario (Roberts et al., 2008). Low available P has been shown to decrease organic grain production in the long-term (Carkner et al., 2020), and limits the productivity of legumes in commercial green manure crops (Thiessen Martens et al., 2021).

Phosphorus is an essential plant macronutrient, as it contributes as a critical structural component of nucleic acids and plays a key role in energy transfer (Marschner, 1995; Grant and Flaten, 2019). Currently, the approved P fertilizer options for organic use are manure and rock phosphate. However, manure is often prohibitively expensive to purchase and transport, especially for large, stockless organic farms on the Canadian prairies where animal manure sources are geographically separated from cropland (Schneider et al., 2019). Moreover, phosphorus in rock phosphate is generally unavailable in the year of application due to its low solubility, especially when applied to calcareous soils with high pH, which is a common characteristic of Canadian organic farms (Martin et al., 2007). Despite the many attempts to increase the availability of rock phosphate through measures such as co-composting, microbial associations, and green manure residue management (Asea et al., 1988; Arcand and Schneider, 2006; Arcand et al., 2010; Ditta et al., 2018; Billah et al., 2020), these methods showed limited effectiveness in improving agronomic response to rock phosphate in organic cropping systems in Canada (Arcand et al., 2010). Additionally, rock phosphate is mined from a non-renewable resource, counterintuitive to the organic philosophy of closing the nutrient cycle on farm (Nicksy and Entz, 2021). Other promising forms of P using unconventional sources are currently being explored on organic farms such as frass from black soldier fly (BSF; *Hermetia illucens*) larvae, anaerobically digested urban food or manure waste, and struvite (NH_4_MgPO_4_·6H_2_O) which is a mineral extracted from municipal wastewater streams or manure (Nicksy and Entz, 2021; Thiessen Martens et al., 2021). However, these options are prohibitively expensive, or not approved for organic use under the current Canadian Standards, for example, Struvite, which is sourced from human waste-water sources (Canadian General Standards Board, 2021). Improving on-farm P uptake/use efficiency of crops and reducing external P imports play a key role for the sustainability of Canadian organic farms.

The Canadian prairies comprises of Alberta, Saskatchewan, Manitoba, and the Peace River region of British Columbia, and represent 50% of all organic land in Canada. This region is known as one of the bread baskets of the world, and wheat is well adapted to grow under cool, wet conditions in central and eastern Manitoba as well as drier, hotter conditions in Saskatchewan and Alberta. Prairie organic farms grow 93% of Canada’s organic wheat, reaching nearly 376 000 hectares in 2020 (Canada Organic Trade Association, 2021). Canada exported approximately 237 000 metric tonnes of organic wheat valued at over $118 000 000 in 2020 (Agriculture and Agri-Food Canada, 2022). Recent premiums for organic grade wheat grain are at 253% of conventional grade wheat grain prices (Organic Biz, 2023). Wheat is often the cash crop in an organic rotation, providing essential economic value to farmers.

Crop selection and breeding for greater P use efficiency (PUE) and P uptake under low soil test P has been proposed as a potential solution to tighten the P cycle on farm (Rose et al., 2013). P management on organic farms brings unique challenges as these farms rely heavily on biologically mediated nutrient supply, that is, mineralizing P from soil organic matter (SOM). Therefore, specific strategies and perspectives are required to optimize P uptake in partnership with crops and reduce off-farm P losses. The development of new crop cultivars that address P challenges on organic farms can contribute significantly to this goal.

### 1.2 Proposal of wheat ideotype to optimize acquisition and utilization on organic farms

**Figure 1:**
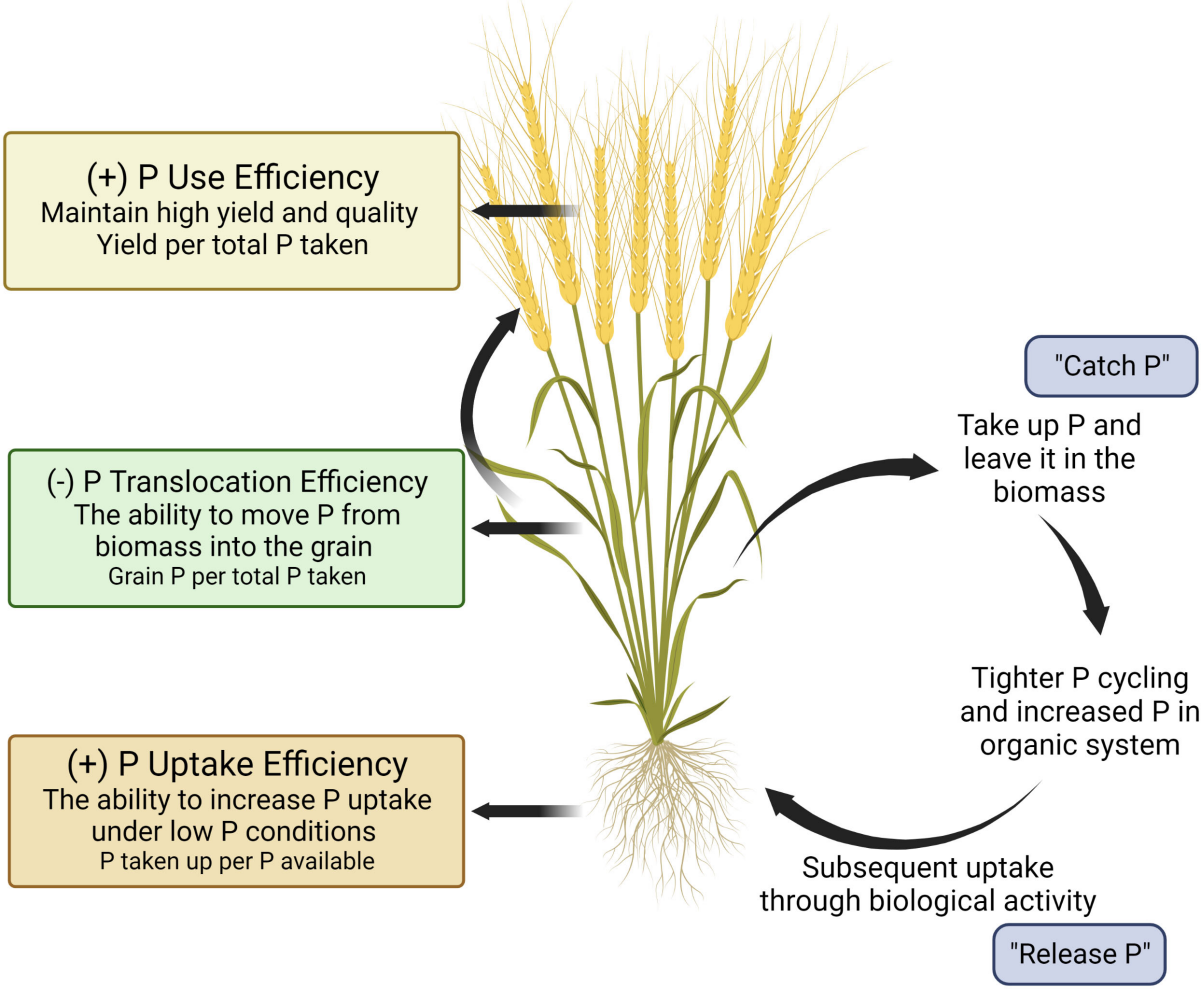
A visual model of the ‘organic ideotype’ of wheat for low phosphorus organic cropping systems. This figure was created with BioRender.

One approach to deploying genetic resources to achieve specific breeding goals is to develop a crop ideotype. An ideotype is defined as “a biological model which is expected to perform or behave in a predictable manner within a defined environment” (Donald, 1968). For common bean and maize cultivars in the Americas, Latin America, and Asia (Lynch and Brown, 2001; Wang et al., 2010; Lynch, 2011; Richardson et al., 2011), an ideotype has been proposed to enhance plant performance under low P conditions that maximizes P uptake through topsoil foraging root architecture, and enhanced soil-P mining strategies. In this paper, we propose a hypothetical wheat ideotype that can maximize P uptake and minimize off-farm P losses via grain P exportation for organic production systems (**Figure 1**). The P-efficient cultivar is characterized by three main features: (i) root topsoil foraging strategies to increase P acquisition, (ii) root mining strategies to mineralize P from organic pools, and (iii) greater P utilisation efficiency (e.g., greater yield per unit P applied) (Richardson et al., 2011). We further propose a reduced translocation of P from shoot biomass into grain should be considered as an important feature for organic production systems. While this concept is not new (Raboy, 2007; Richardson et al., 2011; Rose et al., 2013; Rose et al., 2022; Julia et al., 2018), the importance of P translocation into grain relative to other traits has not been highlighted when considering overall P use efficiency in cropping systems, especially within the context of organic production systems. In addition, to the authors’ knowledge, the implications of lower grain P as a food source beyond the farm gate and as a seed source in organic systems have not been well investigated. The goal of this paper is to explore the potential of incorporating plant traits to increase P acquisition and lower translocation of shoot P into grain P by considering the distinctive nature and needs of organic production systems. Additionally, implications for lower grain P beyond the farmgate and as a subsequent seed source are further explored.

### 1.3 The phosphorus cycle in agroecosystems

The soil P cycle is a complex and dynamic process that involves a range of biological and geochemical transformations influenced by various environmental factors (e.g. soil moisture and temperature). Plants can only take up P in the form of HPO_4_^2-^ (soil pH 4.0-7.2) or H_2_PO_4_^-^ (soil pH >7.2), which are often referred to as plant available P (Pierzynski et al., 2005). Plant available P concentration in soil solution is typically low, less than 1% of the total P in the soil (Pierzynski, 1991). For optimal plant growth, P concentration in soil solution should exceed 0.2 mg P L^-1^. However, a P concentration between 0.2-0.3 mg P L^-1^ indicates the potential for eutrophication in water bodies (Pierzynski et al., 2005; Bacelo et al., 2020), emphasizing the need to understand and manage the P cycle in agroecosystems.

**Figure 2:**
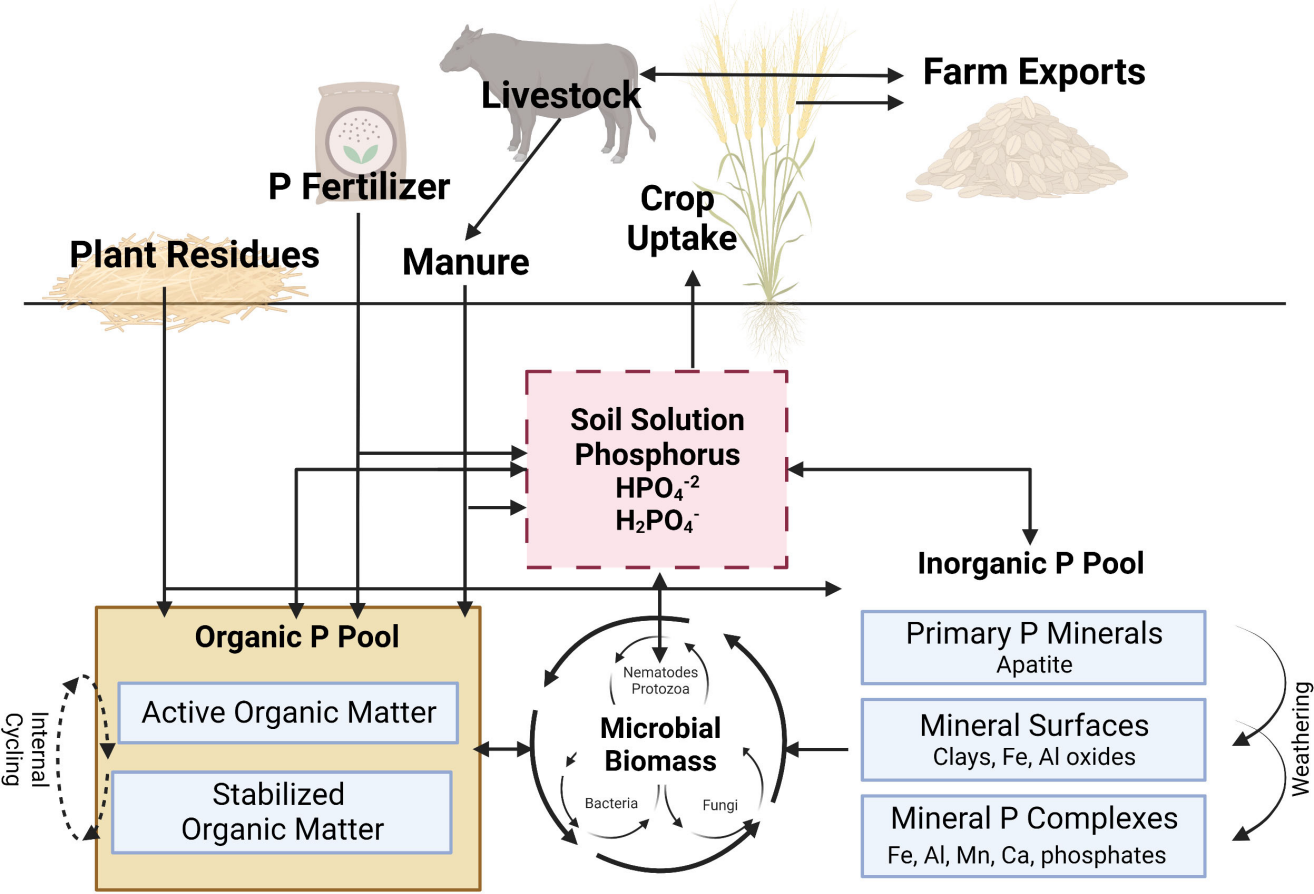
A simplified illustration of the phosphorus cycle in agroecosystems. See text for full discussion of cycling processes. P, phosphorus; Fe, iron; Al, aluminum; Mn, manganese; Ca, calcium. Adapted from Kovar and Claassen, 2005. This figure was created with BioRender.

The majority of soil indigenous plant available P originates from weathering of apatite. In agricultural systems, plant available P pool in soils is also enriched by application of synthetic fertilizers or manure. Once P in soil solution exists as free ions, it can react with dissolved iron (Fe), aluminum (Al), manganese (Mn) in acid soils, or calcium (Ca) and magnesium (Mg) in alkaline soils to form phosphate precipitates (**Figure 2**). Plant available P can also be adsorbed onto clays and the oxides of Al and Fe, taken up by plant roots, or incorporated into the Organic-P pool as microbial infrastructure and/or organic matter (*immobilization*) (Jakobsen et al., 2005; Pierzynski et al., 2005; Drinkwater et al., 2017). Additionally, microorganisms in the rhizosphere may compete with plants for plant available P in the short-term. However, they also have the potential to release P to plants through the process of *mineralization*. Through continuous biological and geochemical reactions, P available to plants and microorganisms are in a constant flux between mineralization/immobilization and adsorption/desorption processes. For the interest of this paper, biological processes (i.e., mineralization/immobilization, root uptake) will be emphasized while geochemical processes (adsorption/desorption, dissolution/precipitation), although extremely important regarding plant assimilation, microbial recycling, and environmental implications, will be given less attention.

### 1.4 The organic P pool and microbial biomass P

Soil organic P refers to P that is bonded in some way with carbon (C). Soil organic P is initially derived from animal wastes and plant residues and is synthesized by soil organisms. Plants and microorganisms take up and assimilate soil solution P which is then bonded to C through phosphorylation (Condron et al., 2005). Soil organic P consists of various forms including orthophosphate monoesters, inositol phosphates (e.g., phytic acid), phosphoproteins, mononucleotides, sugar phosphates, phospholipids, teichoic acid, aromatic compounds, phosphonates, and organic phosphate anhydrides. Many of these compounds exist in the form of highly stable ring structures, making them resistant to hydrolysis and less accessible to plants (Condron et al., 2005; Jones and Oburger, 2011).

It is estimated that organic P pools make up 30% to 80% of soil total P, depending on the cropping systems (Harrison, 1987; Bhattacharya, 2018). The organic P pool is made of dead material from plant, animal, and microbes. The microbial biomass includes bacteria, fungi, algae, protozoa, nematodes, which make up between 2 to 5% of total soil organic carbon (Brookes et al., 1984). The microbial biomass component within SOM is the ‘live’ fraction and responsible for mineralization of nutrients such as P (Jakobsen et al., 2005). The abundance and activity of soil microbes are heavily reliant on C inputs, as well as suitable soil moisture and temperature regimes. Microbial biomass P has been reported to account for 2 to 5% of the soil total P and approximately 10 to 15% of the soil organic P (Richardson and Simpson, 2011).

Mineralization of organic P into plant available P is dependent on the size of the microbial P pool, microbial activity, and the time required for the nutrient pool to renew itself (Oberson et al., 2001). Quantifying the size and turnover rate of microbial P during a crop growing season is challenging due to variations in temperature and moisture content. Using ^33^P isotope tracer in four calcareous soils in Ontario, Schneider et al. (2017) showed that the velocity of microbial P turnover was highest in soil with the lowest available P, despite microbial biomass P concentrations being the same. Using fumigation methodology to assess microbial P content and turnover, Oehl et al. (2001) reported that organic cropping systems had greater microbial biomass P pools and faster turnover rates than conventional systems. Similar results were observed in Canada by Braman et al. (2016). Therefore, the form and rate of P inputs can influence organic P dynamics and availability. Bünemann (2015) reviewed studies on organic P dynamics and reported that the relative contribution of biological and biochemical mineralization of P isotopes to plant available P ranged between 20 and 35% in arable soils, and 50 to 70% in grassland soils. Microbial P dynamics in relation to crop type grown is poorly understood and understudied. To our knowledge, only one study has investigated such a relationship and reported that addition of buckwheat residues with different types and rates of phosphate rock had little effect on the microbial biomass P in an organic dairy farm in Ontario (Arcand et al., 2010).

Numerous studies have demonstrated that plant P availability is also dependent on N availability in the soil system (Lemaire et al., 2021). For example, Briat et al. (2020) illustrated greater P uptake was coupled with greater N supply. Nitrogen mineralization in a cropping system is largely dependent on factors that also influence microbial P mineralization. Therefore, a whole soil system approach is required to understand soil-P availability, especially under organic management, where crops rely heavily on biologically mediated nutrient supply for both N and P. Furthermore, the role of livestock integration into cropping systems also requires attention. The integration of crop-livestock on organic farms in Canada has multiple benefits ecologically and economically (Entz and Thiessen Martens, 2009; Thiessen Martens and Entz, 2011). Additionally, recent arguments have been made that livestock can not only be a source of nutrients (i.e. Manure), but herbivory action has the potential to catalyze nutrient cycling, increasing the microbial pool, and thus creating nutrient pools that cycle more efficiently with less potential for loss (Soussana and Lemaire, 2014; Lemaire et al., 2023). Taken together, achieving efficient P-cycling on organic farms through breeding is important, however, managing the soil system to ensure efficient N-P cycling is equally critical for long-term sustainable production.

### 1.5 Phosphorus uptake in plants and microorganisms

Phosphorus is relatively immobile in soil solution, meaning that plant roots and microorganisms must navigate towards P in the soil for uptake. Roots and microorganisms will encounter new available P pools as they move into unexplored soil that has not been depleted. Phosphorus is transported to microorganisms and plant roots by either mass flow or diffusion. Mass flow involves dissolved P moving towards the plant root/microorganism along with water. Phosphorus transport via mass flow accounts for a very small total P absorbed, even when P concentration in the soil solution is high. Diffusion is the process through which P moves from an area of high concentration to low concentration, accounting for approximately 95% of root P uptake (Kovar and Claassen, 2005). Diffusion is also the main uptake mechanism for microbes such as bacteria and fungi (Jansson, 1988). In plant roots, P uptake from soil is rapid and occurs within cells behind the root tips (Kovar and Claassen, 2005). Phosphorus in the soil solution is much lower than that of the cells within the plant, so P is actively moved across the root membrane by phosphate transport proteins against a concentration gradient (Smith et al., 2003). Phosphate transporters have also been detected in fungi and bacterial organisms (Jansson, 1988).

**Figure 3:**
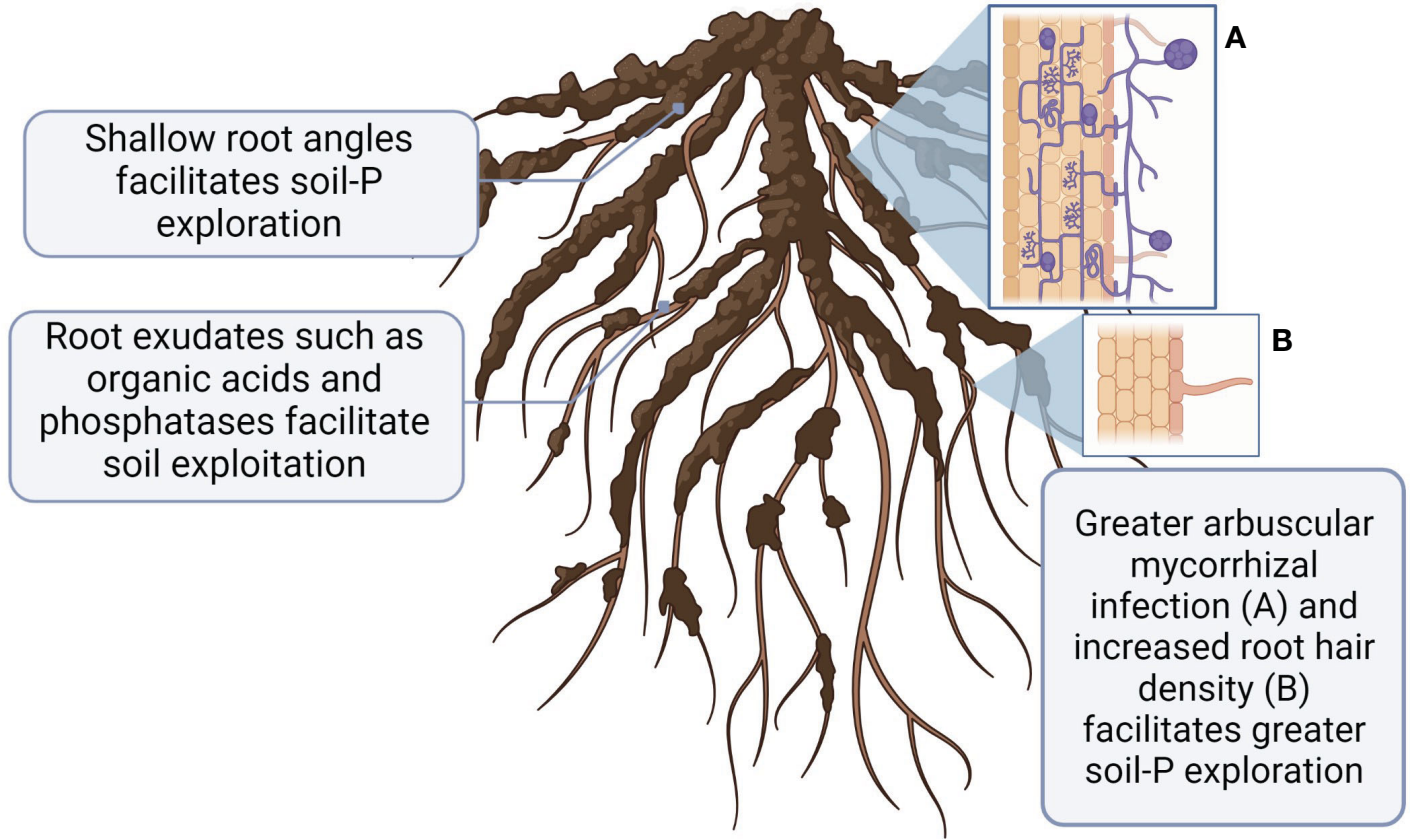
A schematic representation of root characteristics associated with greater P uptake adaptations to low soil P availability. This figure was created with BioRender.

When P in soil solution is taken up by plant root or microorganism, it creates a ‘depletion zone’ adjacent to uptake site (Smith et al., 2003), necessitating constantly increased P access. There are two principal strategies to increase P access as 1) greater soil exploration to new zones of higher inorganic P (via better root growth or association with arbuscular mycorrhizal fungi (AMF), and 2) P exploitation via chemical and biological P transformations to increase more available P uptake (York et al., 2013; Fraser et al., 2015) (**Figure 3**). Plants and microorganisms may employ either P exploration or P exploitation, or a combination of both (Richardson et al., 2011).

## 2 From the ground up: greater P uptake in organic soils

### 2.1 Increased physical exploration of soil

Root traits associated with increased P acquisition by explorative strategies have been extensively studied in wheat under field and greenhouse conditions (Mcdonald et al., 2015; da Silva et al., 2016; Wang et al., 2016; Rabbi et al., 2017; Nguyen and Stangoulis, 2019) and summarized in several review papers (Gahoonia et al., 1999; Lynch and Brown, 2001; Lynch, 2011; Richardson et al., 2011). Greater exploration of the upper soil layer (0-10 cm) by crop root systems is essential due to the generally low P mobility in soils. Root architecture can be divided into the geometric properties that dictate the shape of the root system (root angle, depth, and configuration), and structural properties (pattern of root branching, and growth of root hairs). While some previous works have investigated how root architecture can affect P uptake of bean and corn in response to P deficiency (Lynch and Brown, 2001; Richardson et al., 2011), less research has been done with wheat. The ‘topsoil foraging’ root architecture, which is characterized with wide basal and shallow seminal root angles, has been proposed to maximize soil exploration (Manschadi et al., 2013; Lynch, 2019). Structural characteristics such as greater root hair density, and branching are important for P uptake as they increase root surface area and the volume of soil from which immobile P can be explored, especially under P deficient environments (da Silva et al., 2016). Genotypic differences in root angle have been observed in wheat (Maccaferri et al., 2016; Fradgley et al., 2020; Pariyar et al., 2021), additionally, different root angle responses to contrasting environmental conditions has been widely observed (Manschadi et al., 2013; Chen et al., 2018; Sinha et al., 2018). For instance, greater P uptake in Brazilian wheat genotypes was associated with shallow-angled first and second brace roots (da Silva et al., 2016). Gahoonia et al. (1999) reported that under field conditions barley cultivars with longer root hairs depleted more P from the rhizosphere soil and absorbed more P. Within the same study, wheat genotypes grown in hydroponic media grew longer and greater dense root hairs in response to P deficiency (Gahoonia et al., 1999). Contrary to ‘topsoil foraging’ characteristics, Manske et al. (2000) proposed that wheat genotypes with more developed root systems may better access indigenous P in deeper soil profiles, which may be advantageous to organic farms. However, further investigation is required to determine the optimal root architecture for P acquisition of crops under organic farming systems.

Association with AMF is another strategy plants may use to increase soil exploration capacity. A mutualistic relationship between the host plant and AMF is characterised by a bi-directional nutrient transfer between the two species. The fungus receives carbon substrates from the host plant, and the plant receives nutrients in return (Harrison, 2005). In addition to their roles in P nutrition, AMF also provides other benefits to the host plant such as greater zinc uptake (Gao et al., 2007), lower grain cadmium levels (Singh et al., 2012) and higher water use efficiency (Li et al., 2019). AMF increases P uptake through multiple strategies. They can enhance the host plants’ ability to explore greater soil volume by extending their hyphal network beyond the crops’ P depletion zone (Pepe et al., 2018). They can also stimulate the abundance and activity of bacteria in the rhizosphere that excrete alkaline phosphatases to mobilize organic-P (Zhang et al., 2016; Zhang et al., 2018), thus promoting a highly efficient P-affinity system (Harrison, 2005; Kobae, 2019). Root colonization rates by AMF are affected by the plant-available P levels in soils. For example, under high soil Olsen-P conditions (>50 mg P kg^-1^), plants can access P independently and root AMF colonization decreases (Entz et al., 2004; Schneider et al., 2015). In contrast, under very low soil test P conditions where plant growth is P-limited, root AMF colonization may become parasitic, due to greater carbon acquisition by the fungi relative to the lower P translocation from the fungi to the plant host (Johnson et al., 1997). We confirmed such phenomenon in organic farming systems by showing that root AMF colonization of flax was significantly higher in the low-P organic system relative to the conventional system (Entz et al., 2004).

Genotypic variation in root AMF colonization has been observed in various crop species including wheat (Kirk et al., 2011; Singh et al., 2012; Nahar et al., 2020). However, root AMF colonization depends on both soil environmental conditions and management practices. Production practices used by organic farmers such as cover crops and forages (Lehman et al., 2012; Njeru et al., 2014) and conservative P additions (Schneider et al., 2016) are known to promote root AMF colonization. However, other practices used by organic farmers such as frequent and deep tillage, growing non-mycorrhizal crops, and fallow are known to reduce AMF populations (Gosling et al., 2006). Despite these challenges, satisfactory AMF populations have been found on organic farms (Ryan et al., 1994; Kirk et al., 2011; Chen et al., 2022). At the Glenlea long term rotation study site in Manitoba, Welsh (2007) observed increased diversity and spore abundance of AMF in organic compared to conventional farming systems. Several previous studies have reported a positive relationship between root AMF colonization and crop P uptake efficiency under low P soil conditions (Manske et al., 2000; Nahar et al., 2020). For example, Manske et al. (2000) reported that root AMF colonization rate of 42 wheat genotypes was positively correlated with their P uptake efficiency when grown under P deficient conditions. Similarly, the benefits of AMF to increase P uptake and cause crop growth under low P conditions were frequently reported in other studies (Feng et al., 2003; Wang et al., 2017). Organic farms would benefit from selecting genotypes with a greater affinity to AMF to overcome the P constraints to crop growth. Therefore, high AMF partnership affinity maybe a valuable quality trait to be included in crop breeding program in organic systems, and efforts to increase the capacity for high throughput evaluation of root AMF association are encouraged.

### 2.2 Accessing where you are: soil exploitation

Plants have multiple strategies that can increase soil P availability by immobilizing the less soluble P in the inorganic and organic P pools. Kovar and Claassen (2005) reported that plant roots can exudate organic acids into the rhizosphere to solubilize P from Fe and Al complexes in acidic soils and from Ca and Mg complexes in alkaline soils. However, the organic acid compounds in root exudates can vary with crop species and genotypes, and the mechanisms are not well understood (Kovar and Claassen, 2005). Some plants can also excrete phosphatase enzymes into the rhizosphere to enhance mineralization by breaking down carbon and P ring structures within soil organic matter (Li et al., 2004; Nguyen et al., 2019). The following section will explore the potential of soil P exploitation within the context of genotypic variation among grass species and the potential to select/breed such traits for maximizing crop P acquisition.

By comparing six spring wheat genotypes in a hydroponic nutrient solution study, Akhtar et al. (2016) reported a significant relationship between greater plant P uptake and a decrease in solution pH. Also under hydroponic conditions, Gaume et al. (2001) revealed significant differences in organic acid exudation among maize genotypes in response to P deficiency. It was concluded that developing maize genotypes with increased citric and malic acid in root exudates can be an effective strategy to adapt to low P conditions. Nguyen et al. (2019) investigated wheat root exudates under varying P availability in sandy soil and found that a P-efficient genotype ‘RAC875’ released larger amounts of organic acids in root exudates under P deficient compared to adequate conditions. Metabolomics is a new discipline than can provide direct measurements of biochemical activities present in cells, tissues, or an organism (Saia et al., 2019), and it has the potential to work in tandem with genomics to screen breeding lines for metabolites. For example, metabolomics can detect organic acids and phosphatases present in root tissues between genotypes under P stress (Nguyen et al., 2019). Clearly, the strategies wheat genotypes use to access P under low P conditions varies widely. The challenge for breeders and physiologists interested in enhancing P acquisition and utilization in genotypes will be to identify the most beneficial traits for their specific breeding goals.

Despite low P levels on organic farms, satisfactory yields can still be produced (Martin et al., 2007). Additionally, research in Ontario (Schneider et al., 2017) and Manitoba (Braman et al., 2016) reported higher organic P content in forage-based soils in organic relative to conventional farming systems, which may explain why organic farms maintain acceptable forage yields despite low soil test P. Organically managed soils are sometimes characterized by more abundant and diverse soil microbial communities (Mäder et al., 2002; Braman et al., 2016), which can lead to greater soil nutrient supply due to the increased mineralization capacity. This leads to questioning the relevance of current soil P tests dictating availability on organic farms, due to richer soil microbial communities (Braman et al., 2016), and the potential for biologically mediated P supply (Welsh et al., 2009).

Unpredictable fertilizer response in agroecosystems has led to a recent argument that researchers and practitioners can no longer rely solely on soil tests as a diagnostic tool for crop fertility (Lemaire et al., 2021). The interactions between plant demand and soils, nutrients to each other, and the role microbial communities play in the rhizosphere also needs to be considered (Briat et al., 2020). Bioassay diagnostic tools evaluating crop uptake for plant P nutrition in organic production systems (Carkner et al., 2020), and the creation of the N Nutrition Index (NNI) (Lemaire et al., 2008; Lemaire et al., 2021) have been proposed. Greater understanding of plant-soil interactions and proper diagnostic tools are needed to accurately assess genotypic variation in P uptake.

Given greater biological activity and potentially larger organic P pools on organic farms, increasing cultivars’ capacity to access these pools would reduce the reliance on external P inputs (Sattari et al., 2012; Menezes-Blackburn et al., 2018). However, relying only on the soil organic P pool can lead to organic matter mining and decomposition. Therefore, adequate crop residue return is essential on organic farms (Arcand et al., 2016). The proposed P efficient wheat ideotype considers the unique P dynamics in an organic cropping system and the untapped potential of biologically mediated P supply between the root-soil interface. The ideotype would need to possess root characteristics of soil exploration and exploitation to facilitate greater uptake under low P, organic conditions.

## 3 Challenges of using the correct phosphorus use efficiency indices for screening genotypes

Investigating crop cultivars for phosphorus use efficiency (PUE) has been proposed as an effective way to close the P cycle on organic farms (Vance et al., 2003; Schneider et al., 2019). The term PUE is recognized as a combined effect of: (1) increased acquisition and uptake, and (2) increased P utilization (Vance et al., 2003; Veneklaas et al., 2012; van de Wiel et al., 2016; Cong et al., 2020). The term PUE has been used inconsistently throughout literature, and evaluated using different calculations (Bovill et al., 2013, **Table 1**). High P utilization efficiency is defined as growth/biomass production per unit P uptake and is highly associated with the remobilization of P from old to new tissues. In the last decade, little progress has been made in breeding crops with higher P utilization efficiency (Rose et al., 2011; van de Wiel et al., 2016). To select for greater P utilization efficiency, some breeders have chosen to either evaluate cultivars that can maintain high yields under lower soil-P status or evaluate cultivars that increase yields without increasing fertilizer rates. In organic farming, it is critical to develop cultivars that can maintain high yield and quality under low soil available P status. Therefore, an ideotype for organic farming should maximize soil exploration through better root architecture, increased root hair growth and AMF colonization, and enhance soil exploitation through higher root exudates of organic acids and phosphatase.

**Table 1:** Terms and calculations used to assess phosphorus use efficiency (PUE).

PUE Indicator	Formula	Reference
Agronomic P Use Efficiency	Yield increase/P applied	Hammond et al., 2009
P Use Efficiency (I)	Yield/nutrient supplied	Manske et al., 2001
P Use Efficiency (II)	Shoot biomass/P uptake	Wissuwa et al., 1998
P Use Efficiency (III)	Yield_-P_/Yield_+P_	Mcdonald et al., 2015
P Use Efficiency (IV)	P Uptake Efficiency*Yield/Total Plant P	Manske et al., 2001
P Uptake Efficiency (I)	Total aboveground P/P applied	Osborne and Rengel, 2002
P Uptake Efficiency (II)	Total P accumulated/root weight or length	Liao et al., 2008; El Mazlouzi et al., 2020
P Uptake Efficiency (III)	Total Plant P/P Supplied	Moll et al., 1982 via Manske et al., 2001
P Acquisition Efficiency	Total Plant P/P Applied	Osborne and Rengel, 2002
P Utilisation Efficiency (I)	Grain yield/Total P Uptake	Manske et al., 2002; El Mazlouzi et al., 2020
P Utilisation Efficiency (II)	Shoot dry weight/Shoot P Concentration	Siddiqi and Glass, 1981
P Utilisation Efficiency (III)	P harvest index/grain P concentration	Manske et al., 2001
P Harvest Index	Grain P/Total P	El Mazlouzi et al., 2020
P Utilisation Efficiency (DM)	Shoot Weight/Shoot P	Mcdonald et al., 2015
P Utilisation Efficiency (GY)	Yield/Grain P	Mcdonald et al., 2015
Shoot P Utilisation Efficiency (I)	Shoot biomass/P uptake	Su et al., 2006
Shoot P Utilisation Efficiency (II)	Shoot biomass/P uptake (shoots and roots)	Osborne and Rengel, 2002
Biomass Utilisation Efficiency	Biomass yield/P uptake	Batten, 1992
P Efficiency Ratio (I)	Yield/P Uptake	Jones et al., 1989
P efficiency Ratio (II)	Shoot growth at low P/Shoot growth adequate P	Ozturk et al., 2005
Relative Grain Yield	Yield_-P_/Yield_+P_	Graham, 1984
Root Efficiency Ratio	Total plant P/Root Dry weight	Jones et al., 1992
Apparent Remobilisation of P (%)	Apt_1_ - Apt_2_/Apt_1_ x 100 Apt_1_ = P conc. in shoot at first harvest Apt_2_ = P conc. in shoot at second harvest	Hocking and Pate, 1977
Phosphate Acquisition Efficiency	Shoot_-P_/Shoot_+P_	López-Arredondo et al., 2014

Adapted from Bovill et al. (2013).

Depending on the calculations used, the selection for P efficient genotypes could be vastly different. Many studies have based PUE calculations on early nitrogen use efficiency (NUE) work, which calculated NUE as the ratio of grain N uptake per unit of N available in the soil (Manske et al., 2001; Ortiz-Monasterio et al., 2001; Manske et al., 2002; Manschadi et al., 2013; Mcdonald et al., 2015; Meier et al., 2022). This can be problematic for assessing PUE as phosphorus availability and behavior in soil-plant systems differs markedly from nitrogen. It is imperative that breeders should select the correct PUE measurements as it relates to their goals, and not rely on PUE precedence in the literature. Similarly, redefining the concept of NUE towards integrating soil-plant relationships (Ciampitti et al., 2022) and evaluating genotypes on the basis of effective use of N rather than responsiveness to added N has also been argued (Ciampitti and Lemaire, 2022). Selecting genotypes based on their responses to added P through P acquisition efficiency or relative grain yield is inappropriate for organic production systems. For example, a genotype with poor performance under low P conditions and a greater response to P fertilizer might yield well in conventional systems but would not be desirable for organic farming. In contrast, a genotype that has high uptake potential under low or biologically mediated nutrient supply, yield per unit of P uptake, and low P translocation from the vegetative to reproductive organs would be useful. Wheat cultivars with such traits can take up high amounts of soil native P in the current growing season while releasing P for the following crop when wheat residues are returned (**Figure 1**).

Selecting crop cultivars that can simultaneously increase P uptake and utilization efficiency is a challenge since the two traits are intimately linked. Increasing P uptake, and therefore P in biomass often reduces internal utilization efficiency (Veneklaas et al., 2012). Therefore, it is suggested that different genotypes should be targeted for uptake and utilization to optimize the overall PUE. Ultimately, finding a way to combine both traits in a single genotype needs to be considered (van de Wiel et al., 2016). Comparing cultivars’ aboveground biomass per unit P uptake at anthesis before P translocation from biomass into grain occurs would be beneficial to maximize P uptake and utilization potential.

## 4 The consequences of low translocation of vegetative P to grain P

The phosphorus harvest index (PHI) is defined as the ratio of grain P to total plant P, and it represents the amount of P translocated from the vegetative biomass into the grain. Once P is taken up and used for vegetative growth, the remaining P is stored in the vacuoles (Veneklaas et al., 2012). Depending on P supply, pre-anthesis P uptake can contribute up to 81% of grain P accumulation (Batten, 1992; El Mazlouzi et al., 2020). It has been proposed that selecting crop genotypes with lower PHI would be a beneficial trait to reduce external P inputs (Batten, 1992; Rose et al., 2013; Vetterlein and Tarkka, 2018; Cong et al., 2020). While high protein content in wheat grain is a market premium, greater grain P is not. Grain P is stored mainly as phytate and to a lesser number, chemical compounds including inorganic phosphate, phospholipids, DNA, RNA, and ATP (Rose et al., 2013). Phytate is poorly digested by monogastric mammals, and often becomes a pollutant to waterbodies from livestock and city wastes (Schneider et al., 2019). Reducing grain P could contribute to decreasing off-farm P exportation. For example, Rose et al. (2010) estimated that a 20% reduction in rice grain P would globally reduce P removal from fields by 0.4 Mt per year.

However, would reducing P translocation to grain adversely affect grain yield and quality? The relationships between PHI and yield are consistently weak (Batten and Khan, 1987; Jones et al., 1989; Rose et al., 2011; Mcdonald et al., 2015), indicating that low P translocation may not affect final grain yield. Movement of carbohydrate and P into grain sink is regulated independently, and it is reported that P movement occurs earlier and faster than carbohydrates (Batten and Khan, 1987; Peng and Li, 2005). Currently, cereal crop breeding efforts are mainly focusing on increasing grain yield while maintaining protein (Wang et al., 2003). However, little is known about how decreasing seed P would play a role. Early studies demonstrate that low seed P can be combined with satisfactory protein levels in wheat, legumes, and oilseed rape (Batten and Khan, 1987; Chitra et al., 1995; Lickfett et al., 1999). For instance, Lickfett et al. (1999) reported a significantly negative correlation between phytate and protein in crop grains. In contrast, we recently observed an inconsistent relationship between grain P and protein levels under organic management, due to P deficiency resulting in low grain P, lower yields, and high protein (Carkner, 2023). Grain yield and protein are generally negatively correlated (Iqbal et al., 2016), so lower grain P in combination with lower yield would be expected to result in higher protein. Further research is needed to explore the potential impact on grain quality by reducing P translocation from crop biomass into the grain on organic farms with satisfactory yield conditions.

If an ideotype can produce high yield with low grain P concentration, how will this affect seedling vigour when the grain is used as seed? Will low grain P lead to a reduction in early season vigour due to depleted seed P reserves (White and Veneklaas, 2012)? Crop seedlings rely on P reserves for early growth and root establishment, up to three weeks after germination (Grant et al., 2001; White and Veneklaas, 2012). Many studies have reported greater seedling vigour and increase P uptake due to faster root growth when comparing P-rich seeds with P-poor seeds (Thomson and Bolger, 1993; Rose et al., 2012; Lorts et al., 2020). However, source seeds for experiments are usually from P-depleted soils, and it has been argued that poorer seedling vigour may be an artifact of poor seed quality rather than low P concentration (Julia et al., 2018). Some studies with wheat and rice have also reported that seed size, but not seed P content, influenced seedling and root growth (Derrick and Ryan, 1998; Julia et al., 2018). Additionally, Pariasca-Tanaka et al. (2015) reported significant genotypic by seed-P interactions for seedling vigour in rice, demonstrating that genetic variation may be a tool to manipulate this trait. While average wheat seed P concentration ranges from 3.4-4.5 mg P g^-1^ (Selles et al., 2011), Rose et al. (2013) reported that some genotypes with seed P concentrations as low as 1 mg P g^-1^ did not reduce germination, seedling, vigour, or final grain yield. Finally, Julia et al. (2018) demonstrated that rice seedlings acquire P from outside seed reserves after 2 days after germination. However, their research was conducted in growth media with synthetic P supply. Under organic conditions, where crops rely on biological activity for P nutrition, cold soils in the spring may hinder uptake. Greater understanding of P supply and seedling growth under organic conditions would be valuable to address this issue. Additionally, May et al. (2022) demonstrated that certain cover crops like black medic can increase soil available P over time, which may result in greater seedling nutrition to overcome low seed P reserves. Therefore, with the use of strategic ecological agronomy, there is a potential to select genotypes that can access and store greater P, while translocating less P into the seed for better P cycling. This approach may allow for breeding ideotypes with low seed P levels that do not suffer negative consequences on seedling vigor.

### 4.1 Crop residue potential to increase soil phosphorus availability

After grain harvest, organic farmers often incorporate the remaining crop residue into the soil either in the fall or early spring; this is typical in extensive systems of the Canadian prairies where straw is not collected for animal bedding. The ability of the ideotype to ‘release’ P back into the soil system through mineralization processes may provide a valuable nutrient source for following crops and for building soil organic matter (Arcand; Kucey et al., 1989), or both.

A core component of the organic ideotype is its ability to release (mineralize) P from plant residue, thus increasing P cycling in the rotation and reducing the reliance on external P inputs. The bioavailability of P in crop residue depends on the the amount and forms of P present. For instance, the biomass of wheat residue (excluding roots) has been estimated up to 7.4 t ha^-1^ (Liu et al., 2019). Damon et al. (2014) provided an extensive literature review on residue contribution to P pools in agricultural soils, reporting that average wheat residue P amounts in southern Australian grain cropping systems are 0.4, 1.8, and 5.4 kg ha^-1^, under low, medium, and high productivity scenarios, respectively. Tillage may have an influence on P mineralization. For example, wheat residues immobilized 0.2 kg P ha^-1^ under no-till management, and mineralized 0.4 kg P ha^-1^ under conventional tillage during decomposition (Lupwayi et al., 2007). However, the authors indicated that the amount P that was mineralized was too small to contribute significantly to the following crops’ P fertility. No-till management may also increase P surface runoff during spring snow melt, causing losses to the system (Grant and Flaten, 2019; Liu et al., 2019). Canadian organic farmers typically incorporate a no-till phase within their rotation (Halde et al., 2015), or leave wheat stubble untilled between harvest and time of spring crop seeding the following year. Therefore, it is essential to consider surface P runoff loss when no-till is being employed on organic farms, particularly when P content in crop residule is high.

### 4.2 Implications for environmental protection and human nutrition

Phosphorus loss from agricultural lands poses a serious threat to water quality of Canadian watersheds. Over the last decade, major Canadian lakes such as Lake Winnipeg and Lake Erie have experienced severe algal bloom outbreaks (Liu et al., 2021). When excessive P from synthetic fertilizers or manure are applied to agricultural lands, they are subject to losses and being transported by surface runoff and drainage in variable proportions of dissolved P and particulate P (Hart et al., 2004). In cold climates like Canada, dissolved P loss associated with snowmelt runoff has been identified as the dominant P transport pathway to water bodies (Jamieson et al., 2003). On some organic farms, especially those located where animal manure is plentiful, manure is often used as a primary N and P source for crops. However, due to the relatively lower N:P ratio in livestock manure relative to crop needs, manure application usually results in accumulation of P in soils and further an environmental concern for water quality. On organic farms where animal manures are less available, and hence more expensive, farmers typically use manure only to satisfy the P deficit, relying on legumes to supply N (Thiessen Martens et al., 2021).

While many previous studies have focused on improving farm management practices such as 4R Nutrient Stewardship and tile drainage (Grant and Flaten, 2019) and regulations on manure production and application, the current paper proposes crop genetic variation as a strategy to maximize crop P uptake and use efficiency. The proposed ideotype for organic farming systems will identify the key traits for PUE including 1) increased root architecture, root hair growth and AMF colonization; 2) efficient phosphate remobilisation strategies; and 3) optimizing biomass P uptake while minimizing allocation to reproductive seeds. Thus, the improved P acquisition and utilization of the ideotype on organic farm can contribute greatly to reducing P loss to water bodies by decreasing P inputs from organic sources. The proposed ideotype also addresses the concerns of producers and policymakers as it simultaneously reduces fertilizer/manure costs and environmental risks.

In addition to the environmental issue, improper P management in crop production systems can negatively affect grain nutritional quality and thereby influence human health. Phytate constitutes 60-80% of total P in most crops and dominates the storage form of P in wheat grains (Gupta et al., 2015). Phytate is often considered an antinutritional compound as it strongly binds to micronutrients such as zinc (Zn) and iron (Fe). Low bioavailability of these minerals in cereal grains can lead to deficiencies in human populations who rely mainly on cereal foods for calorie intakes. The phytate to Zn or Fe molar ratio in wheat grain has been generally used to categorize their bioavailability (Bouis and Welch, 2010). Canada is a world-leading wheat producer, and it exports 75% of its wheat products, including to developing countries where people are at high risk of malnutrition (Statistics Canada, 2019). Breeding a wheat ideotype with high PUE and low grain phytate can potentially play an important role in alleviating the global prevalence of micronutrient deficiencies. This can be even more promising for organic farming systems due to the benefits on grain micronutrient accumulation. For example, previous studies from the Glenlea Long-term Rotation Study site showed that wheat produced organically in the perennial rotation had higher Zn than the annual rotation, whereas there was no crop rotation effect when wheat was produced conventionally (Turmel et al., 2009). Further studies are needed to understand the biosynthesis and allocation of phytate throughout the crop life cycle and its influence on bioavailability of micronutrients.

## 5 Conclusion

We propose a hypothetical ‘catch and release’ wheat ideotype that possesses traits facilitating enhanced P uptake (‘catch P’ in biomass) under low-P supply, reducing P translocation from the biomass into the grain and thereby returning P back to the cropping system by way of crop residue (‘release P’).Finally, the ideotype minimizes off-farm harvest removal for organic production systems, which impacts off-farm P pollution in addition to enhancing micronutrient bioavailability as a food source. The ideotype would carry characteristics such as greater root exploration and exploitation strategies designed to interact with soil microbial communities. To select for greater “P use efficiency”, we argue that current indices used for conventional agriculture are not appropriate, and breeders should use greater uptake efficiency, yield per unit P uptake, and P harvest index to evaluate genotypes.

Early seedling vigour is of particular importance to organic farmers because crops need to compete with early season weed competition. Early season weeds have the largest impact on final yield (Mason and Spaner, 2006). Lower seed P may hinder early seedling vigour due to poorer seed nutrition, but this is unclear. Further research examining the impact of lower seed P and genotypic effects on early vigour under organic conditions would be useful.

Lower seed P provides additional benefits of reducing exports off farm, therefore reducing P entering the wastewater system and polluting major Canadian fresh watersheds. However, residue management needs to be considered to avoid P leaching from high P biomass on farm. Lastly, low seed P is beneficial from a nutritional standpoint, as it leads to improved Zn and Fe bioavailability, potentially playing an important role in the nutritional portfolio of developing countries where Zn and Fe deficiency is prevalent. Taken together, our ideotype attempts to address P challenges on organic farms from a systems perspective, incorporating nutrient cycling dynamics, environmental considerations, and nutrition. As we move into a new paradigm of sustainable food production where external nutrients are becoming scarce and an increasing number of people face malnourishment, multi-pronged approaches will be required to address these challenges.

## Data availability statement

The original contributions presented in the study are included in the article/supplementary material. Further inquiries can be directed to the corresponding author.

## Author contributions

MC and ME conceived the idea. MC led writing the manuscript. XG contributed to writing portions of the manuscript and editing. All authors contributed to the article and approved the submitted version.
